# Proteomic-Based Approaches for the Study of Cytokines in Lung Cancer

**DOI:** 10.1155/2016/2138627

**Published:** 2016-06-30

**Authors:** Ángela Marrugal, Laura Ojeda, Luis Paz-Ares, Sonia Molina-Pinelo, Irene Ferrer

**Affiliations:** Medical Oncology Department, Hospital Universitario Doce de Octubre and Centro Nacional de Investigaciones Oncológicas (CNIO), 28041 Madrid, Spain

## Abstract

Proteomic techniques are currently used to understand the biology of different human diseases, including studies of the cell signaling pathways implicated in cancer progression, which is important in knowing the roles of different proteins in tumor development. Due to its poor prognosis, proteomic approaches are focused on the identification of new biomarkers for the early diagnosis, prognosis, and targeted treatment of lung cancer. Cytokines are proteins involved in inflammatory processes and have been proposed as lung cancer biomarkers and therapeutic targets because it has been reported that some cytokines play important roles in tumor development, invasion, and metastasis. In this review, we aim to summarize the different proteomic techniques used to discover new lung cancer biomarkers and therapeutic targets. Several cytokines have been identified as important players in lung cancer using these techniques. We underline the most important cytokines that are useful as biomarkers and therapeutic targets. We also summarize some of the therapeutic strategies targeted for these cytokines in lung cancer.

## 1. Introduction

Lung cancer is one of the most frequent types of cancer worldwide, accounting for approximately 13% of the total cancer diagnoses in the most recent global statistics [1, 2]. Adenocarcinoma, squamous carcinoma, large cell carcinoma, and small cell carcinoma are the four most prominent histological types of lung cancer. The first three classes are collectively named Non-Small Cell Lung Cancer (NSCLC) and they represent 85% of lung cancer cases [3]. In particular, adenocarcinoma is the most often reported subtype of NSCLC in most countries [4].

Lung cancer is characterized by a poor prognosis, with a five-year survival rate of 15%, mainly due to an initial diagnosis at advanced stages of the disease [5]. For this reason, in addition to advances in treatment, the search for diagnostic strategies for early lung cancer detection is very important. Thus, the use of biomarkers is essential for early detection. A biomarker is a measurable indicator of a biological process. There are three different groups of protein biomarkers: diagnostic biomarkers, prognostic biomarkers, and biomarkers that predict the treatment response [6].

Proteomics is the systematic analysis of protein profiles in tissues or cells [2, 7] and is directly related to genomics because proteins are the final effectors of the genes in nearly all situations. Proteins are extremely dynamic molecules whose function is regulated by posttranslational modifications, degradation, and compartmentalization [8]. Therefore, the functional protein concentrations would not always be related to the differential expression of mRNAs. For these reasons, proteomics may contribute to improving our knowledge of cancer, in addition to genomics or transcriptomics [9, 10]. Thus, proteomics is a particularly appropriate tool to research lung cancer because proteomics approaches are much more objective and more precise than current methods of cancer diagnosis and patient stratification, which have been based on the study of tissue specimens by pathologists [10].

In the history of cancer studies, different hallmarks have been described that characterize tumor initiation, promotion, and invasion. Some of these hallmarks are related to tumor cells by sustaining proliferative signaling, evading growth suppressors, activating invasion and metastasis, enabling replicative immortality, inducing angiogenesis, and resisting cell death. Later, four new emerging hallmarks were added from studies of the tumor microenvironment: evasion of immune destruction, tumor-promoted inflammation, genome instability and mutation, and deregulated cellular energetics [11]. Thus, taking the tumor microenvironment into account, inflammation is an important factor in the pathogenesis of cancer [12, 13]. Inflammatory cells can provide growth and survival factors, which contribute to several hallmarks of cancer. Similar to other tumors, it has been reported that chronic inflammation due to pulmonary disorders such as chronic obstructive pulmonary disease (COPD) significantly increases the patients' risk of developing lung cancer [14, 15]. Inflammation is regulated by the tumor microenvironment, which plays an important role in immune suppression or activation and in the epithelial-to-mesenchymal transition [16–18].

The main mediators of inflammation are cytokines, proteins that can be classified as proinflammatory and anti-inflammatory molecules, such as chemokines and growth factors [19–21]. These proteins can modulate different cellular responses, including inflammation, the immune response, apoptosis, and chemoattractant processes [22–25]. Characteristic cytokine patterns have been described in different cancer patients and are related to their prognosis. Therefore, some cytokines are good prognostic biomarkers of cancer [26–28].

We review the cytokines that are good biomarkers for the diagnosis, prognosis, and prediction of treatment responses in patients with lung cancer as well as the cytokines that could act as therapeutic targets and describe the therapeutic strategies based on these targets that are being used in clinic. In addition, we describe some proteomic techniques that are the best tools to study these important molecules. An in-depth analysis of the cytokine patterns using proteomics could provide important insights into clinical purposes.

## 2. Proteomics in Cancer Research

**Figure 1:**
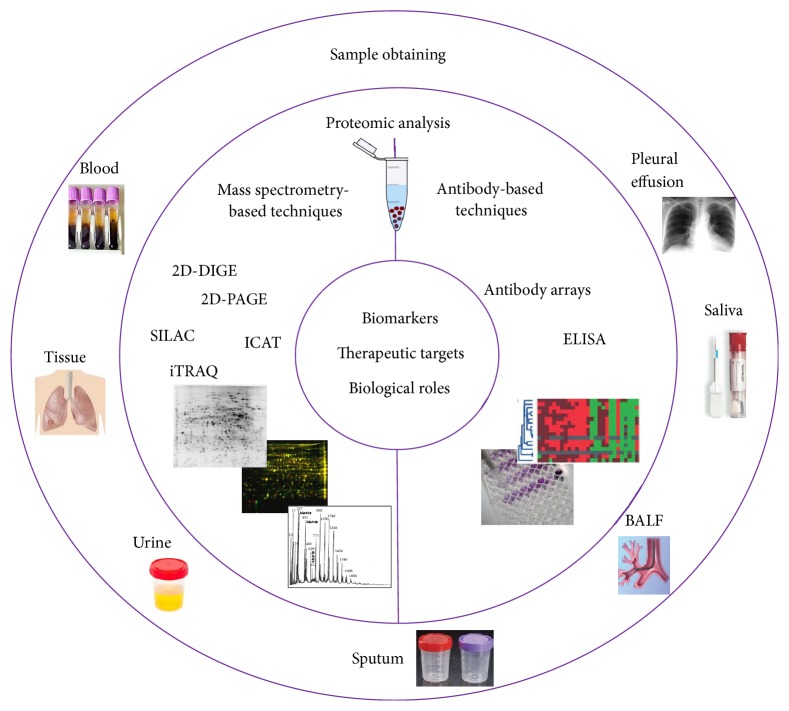
Workflow of the proteomic studies of cancer.

Proteins are the real functional players in cells and define their phenotype [29]. Thus, in some terms, they could provide more precise information about cancer than DNA or RNA. In fact, proteomic analyses also have the ability to quantify the effects of genetic abnormalities related to oncogenesis. Among these quantifiable changes, we highlight the differential expression of proteins encoded by genes with altered DNA copy numbers, splice variants, mutations, deletions, and insertions and regulation by microRNAs or epigenetics. Consequently, proteomics can improve our biological knowledge of cancer and help in the search for new potential therapeutic targets and biomarkers by connecting cancer phenotypes and genomic alterations [30]. The proteomic approaches that are currently used to study cancer and the samples used for this purpose are described next (Figure 1).

### 2.1. Proteomic Techniques Used in Cancer Research

Since the late 90s, the development of high-throughput platforms has allowed researchers to measure thousands of proteins and their modifications. Thus, proteomic assays have become essential tools to decisively detect the molecular patterns in malignant cells, which might be associated with disease evolution or the treatment response [31, 32]. In the last few years, the use of proteomic techniques in cancer research has produced great number of studies [5, 32–36]. The most frequently used proteomic techniques can be divided into gel-based or gel-free approaches, both of which are based on mass spectrometry (MS) and antibody-based techniques.

#### 2.1.1. Gel-Based Techniques-Mass Spectrometry

These proteomic techniques employ two-dimensional gel electrophoresis (2D-PAGE), due to its relatively low cost and high applicability. In this assay, intact proteins are separated in two dimensions. Firstly, previously solubilized and denatured proteins are separated by their isoelectric point. Secondly, proteins are separated by their molecular weight to obtain different protein spots. Later, the resulting spots are analyzed and spots are picked and their peptides are digested for MS identification [32]. This technique allows researchers to study a large number of polypeptides in a single run and to evaluate different gels, making it possible to compare the spot patterns between different conditions, such as affected and unaffected patients. Unfortunately, this technique has limitations, which include low throughput, low sensitivity, and the need for large amounts of clinical material. Moreover, it is difficult to separate very low or very high molecular weight proteins, and there could be some variation among gels. However, progress in this approach has reduced gel-to-gel variation by marking the proteins with fluorescent dyes. Using 2D-DIGE, it is possible to evaluate different samples (such as test, control, and reference) in the same gel following the introduction of Cy3, Cy5, and Cy2 dyes immediately before 2D-PAGE [37].

#### 2.1.2. Gel-Free Techniques-Mass Spectrometry

These methods provide high reproducibility by quantifying proteins in a gel-free setting, which decreases variability and allows researchers to measure complex, labelled, or label-free protein samples [38].

As an alternative to 2D electrophoresis, liquid chromatography separation (LC) can be coupled to MS (LC/MS) to identify the proteins contained in complex biological samples. In this workflow, the molecules resulting from the enzymatic digestion of the samples are separated in a liquid mobile phase by employing a solid stationary phase. Then, the amount of each peptide is quantified. These methods are known as shotgun proteomics and they principally use labelling (with a nonradioactive isotope) and nonlabelling approaches [33].

There are several isotope-based labelling approaches in which* in vivo* metabolic incorporation of the labels is essential. There are three principal techniques, ICAT, iTRAQ, and SILAC, according to the label used. Following trypsinization and subsequent MS analysis, these methods allow researchers to quantify and identify proteins from different samples at the same time. ICAT (isotope-coded affinity tag) employs chemical light or heavy reagents to label and compare pairs of samples. iTRAQ (isobaric tags for relative and absolute quantitation) can analyze up to 8 samples in the same experiment following labelling with fourplex or eightplex reagents [39, 40]. Currently, the most suitable method for quantitative proteomics is SILAC (stable isotope labelling with amino acids in cell culture), mainly due to its robustness, reliability, and easy application [41]. This procedure differentiates two identical cell populations growing in distinct culture media. The “heavy” medium contains amino acids (usually arginine and lysine) substituted with stable isotopic nuclei (^2^H, ^13^C, and ^15^N), whereas the “light” medium includes amino acids with the natural isotope. After a sufficient number of cell divisions, the whole proteome of the cell population growing in heavy medium is labelled, due to the incorporation of heavy amino acids into the newly synthesized proteins. Later, equivalent amounts of heavy and light samples are combined, digested, and analyzed by MS. The different signal intensities from both samples make it possible to quantitatively compare their relative abundances in the mixture, due to the specific masses of the heavy and light amino acids [42, 43]. At present, it is possible to compare up to five different samples in a single experiment based on mixtures of several isotopic forms of arginine and lysine [43]. Unfortunately, the clinical application of SILAC is limited because it cannot be used to directly label tissues or body fluids. However, the super-SILAC method has been recently developed to solve this problem. In this assay, a SILAC standard is generated to represent the clinical sample and is achieved using labelling and a combination of different cell lines to obtain a representation of the tissue or body fluid of interest. In the MS analysis, the SILAC standard is the heavy population, which will be compared to the light population from the clinical sample [44, 45]. Therefore, it is possible to differentiate histological subtypes of cancers and to search for biomarkers for use in other applications due to the precise quantification of human tumor proteomes [46].

The last step in all gel-free techniques consists of mass spectrometry analysis. MS has been widely used for protein and peptide sequencing and identification by measuring the molecular weights and charges (*m/z* ratio) of their ions. Firstly, the samples are ionized by an ion source. The main ionization methods employed are electrospray ionization (ESI), matrix-assisted laser desorption ionization (MALDI), and surface-enhanced laser desorption ionization (SELDI), where the sample is introduced as spray, matrix, or chip, respectively. Later, the ionized samples are injected into a mass analyzer, where the ions are separated according to their* m/z* ratios. Time-of-flight (TOF), Fourier transform, and quadrupole-Q, linear quadrupole-LTQ, and Orbitrap ion traps are the methods that are most frequently utilized for this purpose. If a second mass analyzer is added (tandem mass spectrometry), both proteins and peptides could be identified. Thus, mass spectrometry techniques are commonly used for peptide and protein discovery as a real biomarker application [8, 47].

#### 2.1.3. Antibody-Based Techniques

The search of proteomic profiles using antibodies facilitates the systematic examination of the cancer proteome and evaluation of cancer biomarkers [33].

Enzyme-linked immunosorbent assays (ELISAs) are one of the most frequently used methods to identify proteins in biological samples because they are a financially reasonable screening method that is easy to perform. In simple terms, an ELISA is performed in plates with a capture antibody, which specifically binds to the protein of interest, and a detection antibody linked to an enzyme. The enzyme can transform a substrate into a perceptible and quantifiable signal [48, 49]. Moreover, due to proteomic advances, the levels of many proteins, such as cytokines, can be determined at the same time using ELISA-based protein array technology. In this assay, peptides resulting from previously digested protein samples compete with their identical synthetic peptide (prebound to the ELISA plate) for a specific antibody [50]. This approach does not require the isolation and purification of the protein of interest, although its sequence is essential. Thus, in contrast to MS approaches, it is possible to identify proteins from a damaged or unpurified sample using ELISAs [10].

Antibody arrays are multiplex assays that are able to detect a large number of proteins and compare different groups of samples. In this assay, different antibodies are ordered onto a solid support to which the sample is added. Then, proteins can be detected by a laser scanner using a fluorescence signal. Finally, the binding pattern is correlated with the expression level of each protein [51].

### 2.2. Samples Used in Proteomic Studies

In biological proteomic studies, it is essential to choose the type and number of samples for a proper comparison. It is also important to use well-known model systems and controlled clinical samples. In addition, a large number of samples are needed to obtain statistical power. In cancer, researchers must consider the histological type of the tumor as well as its heterogeneity [52]. Different samples can be used in proteomic studies, including tissue, blood (serum or plasma), urine, and different fluids related to the tissue of interest, such as pleural effusions, sputum, or bronchoalveolar lavage fluid (BALF) for the lung [40, 53].

The majority of cancer research studies use paraffin-embedded, formalin-fixed, or fresh-frozen tissue samples. The limitations of these samples are related to their heterogeneity due to the inflammatory and stromal components and necrotic areas adjacent to islands of tumor cells. The use of tissue microarrays (TMAs) or laser capture microdissection to isolate tissue samples on microscope slides is required to solve this problem [54].

On the other hand, blood is an excellent sample for proteomic analysis due to the ease of obtaining a large amount of sample. Blood can be separated into plasma and serum, which is very useful because the depletion of abundant serum proteins is often necessary for the detection of tumor-specific markers [54]. It is also essential to separate proteins by their molecular weights and characteristics, such as ionic charges, modifications (phosphorylation or glycosylation), hydrophobicity, or hydrophilicity, by chromatographic methods to optimize the search for biomarker proteins in blood [10].

Another type of clinical sample that is particularly appropriate for analyses of tumor proteomes is pleural effusion (PE). PE is the fluid that accumulates in the presence of active disease. PE has a similar protein composition to plasma but is more enriched in tumor-derived proteins due to its proximity to the tumor. Therefore, PE is remarkably helpful in understanding tumor mechanisms and identifying cancer biomarker using proteomic techniques [34].

Urine has also been recognized as a potentially useful sample in nonurogenital diseases because it contains thousands of detectable proteins. These proteins are secreted in a mature and stable conformation. This point, together with the easy and noninvasive collection of a large volume of sample, makes urine a perfect biospecimen for the proteomic identification of cancer biomarkers [32].

Other proteomic studies are based on the proteins included in sputum [55, 56], BALF [5, 57], or saliva [37, 58]. These samples are often used to study nonmalignant conditions, although recent studies have employed them to search for potential lung cancer biomarkers. BALF is particularly useful for accessing cell populations that are in direct contact with lung tumors [57]. Saliva is a useful sample because of its easy accessibility and noninvasive collection and because it contains RNAs and a large amount of proteins [58]. Many of these proteins have been shown to be informative for the detection of oral and systematic diseases, such as lung cancer [37].

## 3. Cytokines as Biomarkers in Lung Cancer

Although several cytokines have been detected as powerful biomarkers, few are currently in clinical use because it is not easy to detect some proteins using noninvasive methods and their applicability may not always be very specific.

Here, we summarize the cytokines used as biomarkers in lung cancer, taking into account the different type of samples collected, blood, PE, BALF, lung tissue and sputum [6, 31, 59], and the type of information provided about the biomarker (Table 1). Several studies indicate the presence of different cytokines in samples of cancer patients compared to noncancer controls [26–28]. These cytokines are used as diagnostic biomarkers for the early detection and determination of the stage of the disease. Some examples of the cytokines detected in serum samples are IL-6, IL-2, IL-8, IL-10, IL-18, IL-13, IL-22, vascular endothelial growth factor (VEGF), tumor necrosis factor-*α* (TNF-*α*), and interferon-*γ* (IFN-*γ*) [26, 27, 60–63]. Moreover, increased levels of IL-6, IL-8, IL-18, and VEGF have been detected in BALF [27]. IL-8 and VEGF are common lung diagnostic biomarkers that have been detected in sputum samples [56]. IL-6, IL-22, and VEGF have also been detected in pleural effusion and lung cancer tissue [64, 65]. Some of these cytokines are good biomarkers with both diagnostic and prognostic value and can predict treatment response. Focusing on prognosis, markers are important for predicting tumor progression. IL-6 overexpression is indicative of inferior survival outcomes in patients with NSCLC, and it is related to the acute phase response and cancer cachexia [66, 67]. Furthermore, high levels of IL-8 and VEGF are related to reduced survival of NSCLC patients [56, 61], and basal levels of VEGF and IL-22 in SCLC patients are associated with a poor prognosis [27, 64, 65, 68–70]. Another cytokine, IL-32, has been recently proposed as lung adenocarcinoma prognostic biomarker, as its overexpression in the tumor tissue correlates with a greater number of lymph node metastases [71]. Although most cytokines that are used as prognostic biomarkers have prooncogenic effects in lung cancer, some of them also have antitumor effects. In this sense, IL-37 is expressed at lower levels in the tumor tissues of patients with NSCLC, and it correlates with poorer overall survival compared to patients with high IL-37 expression [72]. Low IFN-*γ* levels are related to a shorter survival due to a lower lymphocyte count, indicating that some cytokines have important roles in the immune responses that protect against tumor formation [66].

On the other hand, as indicators of the treatment response, biomarkers can provide information about drug susceptibility, toxicity, and the clinical outcomes. The pleiotropic role of cytokines in the tumor microenvironment makes it difficult for cancer therapies to always be efficient. Cancer cells develop resistance to chemotherapy and targeted therapies through several mechanisms. In this sense, it has been shown that cancer cells can secrete cytokines that help them evade death induced by several anticancer drugs through the activation of tumor-promoting pathways and the induction of the secretion of other cytokines and growth factors. These molecules are also implicated in antiapoptotic mechanisms, vessel formation, tumor growth, and metastasis [73]. In lung cancer, it has been reported that patients with IL-6 overexpression have a poor response to chemotherapy [66, 67], which is important because IL-6 is administered in combination with cancer treatments because of its ability to induce platelet production [72]. In addition, although the increase in TNF-*α* expression does not have a demonstrated prognostic value, its overexpression in chemoresponsive patients has been used as a biomarker for predicting the treatment response because high levels of TNF-*α* indicate that the patients are sensitive to the treatments [60]. Moreover, the high levels of IL-2 are related to a good chemotherapeutic response in NSCLC patients [63].

Finally, it is known that some polymorphisms can act as genetic biomarkers. An association between some cytokine gene polymorphisms and the risk of developing lung cancer has been described. Variations in the cytokine protein levels resulting from polymorphisms have been investigated, and the conclusions in several meta-analyses are controversial [74–76]. Nevertheless, it has been reported that IL-10 and IL-6 polymorphisms increase the level of these proteins in serum, which correlates with higher number of cases of lung cancer. In the case of IL-10, the alleles IL-10-1082G, IL-10-819C, and IL-10-592 have been observed in lung cancer patients, suggesting a predictive value [74]. A recent study revealed that two IL-10 polymorphisms (-592C/A and -819C/T) show a significant association with the risk of developing lung cancer. In contrast, patients with the third polymorphism analyzed (-1082G/A) did not present susceptibility to this type of tumor [77]. Related to IL-6, several researchers agree that IL-6-174G/C polymorphism in the promoter region has prognostic value because NSCLC patients with G carrier genotypes (GG/CG) show lower overall survival compared with CC genotype carriers [76, 78].

## 4. The Roles of Cytokines in Lung Cancer

Cytokines can be secreted by tumor and stromal cells in the tumor microenvironment and they can function in an autocrine and/or paracrine manner. Although cytokines are important factors that preserve the correct function of the organism, they can act as tumor-promoting or tumor-suppressor molecules in the context of neoplasia [28, 79]. Proteomic tools, such as ELISA or antibody arrays, are currently used to characterize the signaling pathways activated by cytokines in cancer. These approaches are making it possible to elucidate the roles of cytokines in lung cancer.

The increased levels of cytokines in cancer patients indicate their possible functional roles in tumor progression [61, 64, 70, 80–89]. Previously, we have described several cytokines that are used as lung cancer biomarkers (Table 1). Some of these cytokines (IL-6, IL-8, IL-10, IL-22, VEGF, TGF-*β*, and TNF-*α*) have also been studied to determine their role in lung cancer and are described in comparison to other cytokines next. Others, such as IL-2, IL-13, IL-18, and IFN-*γ*, require further in-depth study to determine their roles in lung cancer. Other cytokines that have not yet been shown to function as lung cancer biomarkers (TGF-*β*, IL-17, IL-32, IL-7, and IL-37) can play important roles in this disease, but further studies are required to determine whether they can act as biomarkers.

### 4.1. Prooncogenic Cytokines

There is evidence that some tumor cells may be able to use cytokines as autocrine growth factors and thereby promote tumor growth. Until now, most of the interest in cytokines in lung cancer has focused on IL-6, a proinflammatory cytokine that is upregulated in lung cancer patients and correlates with decreased cancer survival [61, 82, 83, 90–92]. It has been shown that IL-6 displays carcinogenic effects through the activation of the STAT3 pathway [84, 85, 93, 94]. STAT3 activation results in the secretion of malignant pleural effusion proteins and VEGF upregulation in patients' samples as well as increased cell colony formation in soft agar and tumor formation in nude mice [67, 85]. Moreover, the cell survival effect of STAT3 can limit the overall drug response to some lung cancer treatments, such as Erlotinib. Cells treated with Erlotinib exhibit changes in gene expression and the posttranslational regulation of secreted proteins, including IL-6, which is secreted from Erlotinib-treated cells at higher levels. Moreover, IL-6 triggers STAT3 activation, making the Erlotinib-treated cells more resistant to the treatment. STAT3 has well-known effects on cell growth, angiogenesis, immune system evasion, and the prevention of apoptosis [95].

Another prooncogenic cytokine is IL-22, which is a member of the IL-10 family. Its receptor (IL-22-R) is overexpressed in the lung of cancer patients and it is related to poor prognosis [64, 70]. It has been reported that IL-22 overexpression in lung cancer cells protects the cells from apoptosis by activating STAT3, Bcl-2, and Ccl-xL and inactivating ERK1/2 [64].

IL-8 is a proinflammatory chemokine that has autocrine and paracrine functions in lung cancer cells. It contributes to cancer progression, invasion, and metastasis because of its angiogenic and mitogenic properties [96]. IL-8 activates several oncogenic signaling pathways, such as the PI3-K, Ras/MAP-K and Jak/STAT pathways, which produce protumorigenic effects in many cancer types [97].

VEGF and its soluble receptors (VEGFR-2 and VEGFR-3) are expressed in some NSCLC cell lines [98]. VEGF and VEGFRs mediate angiogenesis, which has an important role in cancer progression because it modulates the chemotaxis and migration of endothelial cells [99]. Some signaling pathways that are commonly associated with cancer are activated by VEGF, such as the PI3-K, MAP-K, and STAT3 pathways [100].

Transforming growth factor-beta (TGF-*β*) is a pleiotropic cytokine involved in cancer progression through the PI3-K and MAP-K pathways [101]. TGF-*β* downregulates the epithelial marker E-cadherin and promotes the upregulation of N-cadherin and fibronectin, triggering the epithelial-to-mesenchymal transition and increasing the migratory potential of NSCLC cell lines [102].

IL-17 is another proinflammatory cytokine that is produced by T helper cells, which plays an important role in lung cancer development and the innate and adaptive immune responses in Lewis Lung Carcinoma (LLC) [86]. It has been reported that IL-17 promotes the expression of VEGF, MMP-2, MMP-3, and TNF-*α*, which are proangiogenic molecules. On the other hand, IL-17 increases the level of IL-6 and IL-8 in NSCLC cell lines and activates the STAT3 signaling pathway, mediating tumor angiogenesis [103, 104].

Finally, IL-32 plays an important role in the tumor microenvironment by inducing the secretion of inflammatory mediators, such as TNF-*α*, IL-1*β*, IL-6, IL-8, IL-18, and MIP-2, which are related to invasion and metastasis. In NSCLC, IL-32 transactivates the nuclear transcription factor NF-*κ*B, which upregulates the expression of matrix metalloproteinases (MMP-2 and MMP-9), increasing the invasion of tumor cells [71].

### 4.2. Antitumor Cytokines

Although most cytokines have prooncogenic effects, there is some evidence regarding the antitumor roles of cytokines in lung cancer, which are related to inflammation and the immune system. IL-7 signaling is required to induce an immune response in a lymphopenic mouse model [105]. It has been shown that lymphopenia induces IL-7 secretion and the subsequent proliferation of T cells, antagonizing immune suppression [88, 106]; however, more studies are needed to clarify the detailed role of IL-7 in the induction of the antitumor effects.

IL-37 is a member of the IL-1 family and although it has been described as a suppressor of immune responses and inflammation, some studies have revealed that it has a protective role against cancer progression [107]. In this sense, it has been reported that lL-37 could play an inhibitory role in NSCLC as it has inhibitory effects on tumor growth* in vivo* by decreasing tumor angiogenesis. A high level of IL-37 correlates with a lower level of VEGF in lung cancer cell lines and reduced microvessel density in NSCLC patients [89].

### 4.3. Context-Dependent Cytokines

Some cytokines have two opposite effects in lung cancer progression, according to the molecular context, which is the case for IL-10 and TNF-*α*.

In some cases, IL-10 improves the metastatic capability of lung tumor cells by increasing the vascular density in the primary tumor and increasing the resistance of lung tumor cells to apoptosis by activating STAT3 pathway [87]. On the other hand, IL-10 has an important role in immunosuppression and cell-mediated immunity because it induces the production of regulatory T cells, which can induce immunosuppression and reduce the number of IFN-*γ* secreting cells [108]. Furthermore, IL-10 secretion could result in the deactivation of macrophages, which are important promoters of tumor progression and neovascularization; IL-10 secretion could also decrease angiogenesis by downregulating VEGF [109].

TNF-*α* is another context-dependent cytokine. Although it has been reported that TNF-*α* decreases lung adenocarcinoma cell death [110] and promotes angiogenesis and invasion, there is a positive correlation between the TNF-*α* levels and the chemoresponse [60]. Doxorubicin treatments induce TNF-*α* expression [111]. Therefore, TNF-*α* can trigger cell apoptosis in the context of chemotherapy.

## 5. Cytokines in Lung Cancer Therapy

Immunotherapy, a tool for the treatment of malignancies that changes or stimulates the host immune system, has become a promising approach for cancer therapy [112, 113]. Traditionally, cytokine therapy has had a basic role in human cancer immunotherapy. In 1986, IFN-*α* (Peg-Intron®) was approved by the US Food and Drug Administration (FDA) for hairy cell leukemia therapy. IL-2 (Proleukin®) was approved by the FDA in 1992 and has been used as a single agent to promote endogenous antitumor immune responses for the treatment of metastatic renal cell carcinoma and metastatic melanoma [114]. In 1995, IFN-*α* was ratified as the first immunotherapy for adjuvant treatment of stage IIB/III melanoma [115].

Focusing on cytokine therapies, we can distinguish four options in lung cancer: decreasing cytokine expression in tumor cells, the use of cytokines as a treatment alone or the use of cytokines with other immunotherapies, and the use of endogenous cytokines to provide an advantage to immune system homeostasis [116].

On one hand, treatments based on a decrease in cytokine expression are commonly used. An excellent example of a treatment that reduces cytokine expression is Siltuximab (CNTO 328), an anti-IL-6-chimeric (murine-human) monoclonal antibody. Because IL-6 is involved in the pathophysiology of various solid tumors, such as lung cancer, the clinical use of this antibody has been analyzed in different contexts [117]. This treatment was evaluated in patients with EGFR-refractory or EGFR-resistant NSCLC, as well as in patients with other solid tumors, in Phase I/II study. However, the monotherapy of Siltuximab did not show clinical activity, although further studies with more patient samples should be performed [118]. In the same field, Belagenpumatucel Lucanix® is a whole-cell vaccine that decreases the expression of TGF-*β*2, which is its immune target. This cytokine leads to immunosuppression in lung cancer. Thus, its inhibition is related to a better prognosis in NSCLC patients [119]. Important results in Phase II trial in stage II–IV NSCLC patients showed a dose-dependent difference in survival for the groups treated with the higher doses of Belagenpumatucel-L [120]. Therefore, a placebo-controlled, randomized, Phase III trial in stage III or IV NSCLC patients was performed. When the overall survival of patients treated with the vaccine was compared, improved survival was observed in patients who were previously treated with chemo- or radiotherapy. These results are promising, although more studies are warranted [121].

On the other hand, the use of cytokines alone as a treatment is an excellent option. In some cases, TG4010, MUC-1 antigen-specific liposomal vaccine with the IL-2 gene [122], increases the levels of this cytokine and has been studied in different trials for lung adenocarcinoma. In combination with first-line chemotherapy, first Phase II trial in patients with advanced NSCLC showed the effectiveness of the treatment in patients with a normal number of activated natural killer cells, which improved their outcomes [123]. Based on these results, later Phase IIB trial showed that TG4010 enhanced the effect of chemotherapy in patients with advanced NSCLC [124], which led to Phase IIB/III trial. In these patients, the progression-free survival was improved when they were treated with TG4010 and chemotherapy [125]. Currently, Phase III part of the trial is ongoing.

As mentioned above, the third possibility of cytokine therapy in lung cancer is based on a combination of cytokine therapy with other immunotherapies. In this field, the most relevant clinical study includes the use of cytokines with Adoptive Cellular Therapy (ACT). This therapy consists of the transfusion of *γδ* T cells, natural killer cells, or Cytokine-Induced Killer (CIK) cells to the patients. *γδ* (V*γ*9V*δ*2) T cells are effector cells for immunotherapy that can secrete cytokines and display cytotoxic activity. Due to problems with the* in vivo* expansion of *γδ* T cells, IL-2 has been required to stimulate their proliferation [126]. Based on this improvement, Phase I trial has been performed in patients with advanced or recurrent NSCLC and showed that the *γδ* T cell treatments were viable and safe in this group of patients [127]. Similarly, cytokines are useful in NK cell therapy. IL-15 and hydrocortisone were used to activate and expand these cells* in vitro* and a clinical trial showed that allogeneic NK cells in combination with chemotherapy were safe and potentially clinically effective [128]. CIK cells are cytotoxic T lymphocytes with powerful antitumor activity that control and enhance the immune function of cancer patients [129]. Several studies have proved that CIK cells treatment improves the responses of NSCLC patients treated with chemotherapy, with a higher overall survival, clinical response rate, and T lymphocyte responses. To this end, supplementation with exogenous IL-2 or IFN-*γ* is required for the* in vitro* culture of CIK cells, suggesting the essential role of cytokines in this immunotherapy [130].

Finally, it should be noted that immune checkpoint inhibitors are promising lung cancer therapies that promote immunologic homeostasis through endogenous cytokines. The PD-1 (programmed death-1) signaling pathway is a receptor expressed on activated T cells, and its ligands, PD-L1 and PD-L2, are produced by stromal and cancer cells [131]. The activation of PD-1 following binding to its ligands promotes adaptive immune resistance [132]. PD-L1 overexpression has been noted in several cancer types. Therefore, monoclonal antibodies targeting PD-1 or PDL-1 have shown activity against these tumors [133, 134]. One of these monoclonal antibodies is Nivolumab, a human monoclonal IgG4-kappa antibody against PD-1. In randomized Phase III study, Nivolumab promoted a superior overall survival, response rate, and progression-free survival for NSCLC patients compared to Docetaxel [135]. Nivolumab has obtained regulatory approval (FDA and EMA) as a first-line, standard, platinum-based chemotherapy for NSCLC progression. Another PD-1 blocking antibody is Pembrolizumab; its activity has been studied in Phase I trial and it showed antitumor activity in advanced NSCLC patients [136]. Therefore, Pembrolizumab has been approved by the FDA as therapy for advanced or metastatic NSCLC patients [137]. Based on this achievement, other studies, such as Phase II/III randomized KEYNOTE-001/010 trials, were performed. These studies focused on previously treated PD-L1-positive NSCLC patients. The trial showed an improved overall survival, progression-free survival, and response rate of the patients treated with Pembrolizumab compared to those treated with Docetaxel [138]. Further studies are ongoing, such as Phase III KEYNOTE-042 study, where Pembrolizumab is being compared to platinum-based chemotherapy in NSCLC patients expressing PD-L1 [137]. Based on these promising results, different studies of PD-1 inhibitors are ongoing. However, PD-L1 inhibition is also another excellent strategy. Atezolizumab, a human Ig-G1 antibody targeting PD-L1, has been analyzed in NSCLC patients. In this randomized Phase II trial, Atezolizumab produced an increase in the survival of previously treated patients compared to Docetaxel. The results are most obvious in patients with high expression of PD-L1 [139]. In addition to Atezolizumab, Avelumab and Durvalumab are PD-L1 blocking antibodies that are actually in Phase III trials in NSCLC patients [140].

## 6. Final Remarks

Cytokines are dynamic molecules that can regulate cellular functions and homeostasis in several types of tissues. In a neoplastic context, they can act as modulators of the initiation and progression of the disease due to their abilities to activate several signaling pathways implicated in tumor formation and metastasis. Their overexpression in several cancer types makes cytokines significant candidate biomarker molecules. Proteomics is the current tool used to study the whole proteome in several model systems. It will be useful to identify new biomarkers and study the effects of different proteins on cancer development. In this sense, proteomic approaches have been widely used to identify new cytokines related to lung cancer and they set the stage for the identification of new biomarkers and development of new treatments using these proteins as therapeutic targets. Due to the poor prognosis of lung cancer, it is important to continue to study the biology of this disease, and proteomic studies of cytokines have been widely used for this purpose. Further studies are needed to identify new biomarkers and to understand their roles in lung cancer, as they can act as new targets for the treatment of early stages of tumor progression.

## Figures and Tables

**Table 1:** Cytokines used as different types of lung cancer biomarkers.

Cytokine	Sample	Current purpose as a biomarker	Current well-known function in lung cancer
IL-6	Blood, BALF, and pleural effusion	Diagnostic, prognostic, and predicting the treatment response	Prooncogenic
IL-8	Blood, BALF, and sputum	Diagnostic and prognostic	Prooncogenic
VEGF	Blood, BALF, sputum, pleural effusion, and tissue	Diagnostic and prognostic	Prooncogenic
TNF-*α*	Blood	Predicting the treatment response	Prooncogenic and antitumor, depending on the context
IL-2	Blood	Prognostic and predicting the treatment response	Not yet determined
IL-18	Blood, BALF, and sputum	Diagnostic	Not yet determined
IL-10	Blood	Diagnostic	Prooncogenic and antitumor, depending on the context
IL-13	Blood	Diagnostic	Not yet determined
IL-22	Blood	Diagnostic and prognostic	Prooncogenic
IFN-*γ*	Blood	Diagnostic and prognostic	Not yet determined
IL-32	Tissue	Prognostic	Prooncogenic
IL-37	Tissue	Prognostic	Antitumor
