# Corrigendum to “Proteomic-Based Approaches for the Study of Cytokines in Lung Cancer”

**DOI:** 10.1155/2018/1404780

**Published:** 2018-07-08

**Authors:** Ángela Marrugal, Laura Ojeda, Luis Paz-Ares, Sonia Molina-Pinelo, Irene Ferrer

**Affiliations:** ^1^Medical Oncology Department, Hospital Universitario Doce de Octubre and Centro Nacional de Investigaciones Oncológicas (CNIO), 28041 Madrid, Spain; ^2^University of Seville, Seville, Spain

In the article titled “Proteomic-Based Approaches for the Study of Cytokines in Lung Cancer” [1], there was a missing affiliation for the third author. The corrected authors' list and affiliations are shown above.
